# Antitumor Effects of Natural Bioactive Ursolic Acid in Embryonic Cancer Stem Cells

**DOI:** 10.1155/2022/6737248

**Published:** 2022-02-16

**Authors:** Dong Young Kang, Nipin Sp, Kyoung-Jin Jang, Eun Seong Jo, Se Won Bae, Young Mok Yang

**Affiliations:** ^1^Department of Pathology, School of Medicine, Institute of Biomedical Science and Technology, Konkuk University, Chungju 27478, Republic of Korea; ^2^Pharmacological Research Division, National Institute of Food and Drug Safety Evaluation, Osong Health Technology Administration Complex, Cheongju-si 28159, Republic of Korea; ^3^Department of Chemistry and Cosmetics, Jeju National University, Jeju 63243, Republic of Korea

## Abstract

Embryonic cancer cells (CSCs) could cause different types of cancer, a skill that makes them even more dangerous than other cancer cells. Identifying CSCs using natural products is a good option as it inhibits the recurrence of cancer with moderate various effects. Ursolic acid (UA) is a pentacyclic triterpenoid extracted from fruit and herbal remedies and has known anticancer functions against various cancer cells. However, its potential against CSCs remains uncertain. This study was planned to examine the induction of cell apoptosis by the UA. For cell signaling studies, we performed experiments, which are real-time qPCR and immunoblotting. Also, various cellular processes were analyzed using flow cytometry. The results raised a barrier to cell proliferation by the UA in NTERA-2 and NCCIT cells. Morphological studies also confirmed the UA's ability to cause cell death in embryonic CSCs. Examination of cell death importation showed that the UA formed the expression of the iNOS and thus the cell generation and mitochondrial reactive oxygen generation, which created a reaction to cellular DNA damage by raising the protein levels of phospho-histone ATR and ATM. In addition, the UA created the binding of the G0/G1 cell cycle to NTERA-2 and NCCIT cells, improved the expression levels of p21 and p27, and reduced the expression levels of CDK4, cyclin D1, and cyclin E, confirming the UA's ability to initiate cell cycle arrest. Finally, the UA created an internal mechanism of apoptosis in the embryonic CSC using BAX and cytochrome c regulation as well as the regulation of BCL-xL and BCL-2 proteins. Therefore, UA could be the best candidate for targeting CSCs and thus suppressing the emergence of cancer.

## 1. Introduction

Stem cells are known to differentiate into all types of tissues. Embryonic stem cells can differentiate all types of tissue present in the human body. However, embryonic cancer stem cells (CSCs) have these properties as well as unrestricted proliferation, making them more dangerous than other types of cancer cells [1, 2]. Embryonic CSCs have the potential to differentiate into diverse cancers, such as lung, colon, and breast cancers [3]. Therefore, testing for anticancer drugs relies not only on their capacity to identify cancer cells but also on their capacity to cause cell death in CSCs.

Cancer patients also suffer from the side effects of chemotherapeutic drugs [4, 5]. Cancer treatment using natural ingredients is a better option because of its ability to target targeted therapies with fewer side effects compared to chemotherapeutic drugs [6, 7]. CSCs cause cancer recurrence and promote tumorigenesis. External stimuli or alterations in gene expression may lead to CSC activation by angiogenesis, cell proliferation, and suppression of apoptosis, eventually resulting in tumor and thus metastasis [8]. Cancer treatments that use traditional medicine reduce the size of the tumor, but CSCs are not resistant to conventional medicine. Targeted treatment against CSCs can prevent tumor growth and eliminate the risk of cancer recurrence [9]. Ursolic acid (UA) is a pentacyclic triterpenoid isolated from medicinal herbs and fruits with medicinal and biological functions [10]. It has various anticancer properties such as induction of apoptosis, suppression of angiogenesis, inflammatory responses, antioxidation, and tumor metastasis. In contrast, molecular cell signaling is primarily linked to anti-inflammatory effects using proinflammatory cytokines such as IL-1*β*, IL-17, IL-7, and TNF-*α* or cyclooxygenase-2 and nitric oxide synthase via nuclear factor-*κ*B, a key component involved in the inflammatory response [11]. Studies on UA have illustrated the activation of cell cycle arrest and inhibition of cell growth by inducing the intrinsic and extrinsic pathways of apoptosis *in vitro* and *in vivo* in gastric cancer [12] and breast cancer [13] cells. In non-small cell lung cancer cells (NSCLC), the UA has been found to inhibit autophagy and suppress TGF-*β*1-guided EMT caused by the regulation of integrin *α*V*β*5/MMP signaling, which is considered to be the reason for its anticancer activity in NSCLC cells [14–16]. The UA has also been shown to cause cell death and reduce tumor growth by regulating autophagy-related autophagy 5-dependent autophagy in cervical cancer cells [17]. However, the role of the UA in embryonic CSCs remains to be unclear, and it would be interesting to determine whether UA could target CSCs.

Reactive oxygen species (ROS) play an important role in tumorigenesis as they cause DNA damage leading to a DNA damage response (DDR) mechanism. Often, prolonged stress leads to ROS generation, resulting in double-strand DNA fragmentation, genomic instability, and heterozygosity loss. In response to this situation, DDR activates a DNA-altering method against aberrant DNA [18, 19]. This DDR induction initiates the activation of DNA-dependent protein kinase (DNA-PKcs), Rad3-related (ATR), and ataxia-telangiectasia mutated (ATM) [20, 21]. The ATM signaling activates the expression of tumor protein 53 (p53) in response to DNA damage leading to cell cycle arrest, DDR, and apoptosis mechanisms [22]. The anticancer drug that produces ROS generation can cause increased DNA damage leading to DDR activation due to increased signaling of the ATM/ATR signaling pathway.

The major checkpoints for tumorigenesis are uncontrolled cell cycle progression, the inability to induce apoptosis, and the suppression of tumor suppressor genes. Disruption of cell division or cell cycle stages can lead to uncontrolled cell division, leading to tumorigenesis. Cyclin-dependent kinases (CDKs) are considered to be important regulators of the cell cycle, while CDK inhibitors can regulate cell cycle continuity and prevent normal cells from developing cancer. In the event of DNA damage, the CDK inhibitor, p21 (p21^WAF1/Cip1^), acts as a tumor suppressor in response and stimulates the cell cycle [23]. It is also known as a master effector of the tumor suppressor pathways due to its ability to inhibit p53-independent cell proliferation [24]. The proteins p21 and p27 (KIP1) have the ability to inhibit CDK proteins and cyclin proteins such as cyclin D1; therefore, their anticancer activity depends on the development of p21 or p27 expression and the suppression of cyclin and CDK proteins [25]. Prolonged cell cycle arrest results in cell death, that is, apoptosis. When normal cells cannot stimulate DNA repair or apoptosis in response to stimuli such as mutations, tumorigenesis occurs. Loss of ability to induce apoptosis is an important factor in tumorigenesis, and this technique may be used in anticancer treatment [26, 27].

This study demonstrated the induction of apoptosis by the UA in NTERA-2 and NCCIT embryonic CSCs, and the molecular mechanism underlying apoptosis induction by UA in embryonic cancer stem cells was also analyzed.

## 2. Materials and Methods

### 2.1. Cell Culture

Embryonic carcinoma cells (NTERA-2 and NCCIT cells) were bought in the Korea Cell Line Bank (Seoul, Republic of Korea). NTERA-2 cells were maintained with DMEM, and NCCIT cells were cultured with RPMI-1640 media containing 10% FBS and 1% penicillin at 37°C in 5% CO_2_. At grown up to 80% confluence, the cells were nontreated or treated with UA and then further cultured for 24 h.

### 2.2. Reagents and Antibodies

Fetal bovine serum (12483–020), RPMI-1640 medium (11875–093), 0.05% trypsin-EDTA (25300–054), and penicillin-streptomycin (15140122) were purchased from Gibco (Thermo Fisher). DMEM medium (LM001-51) was purchased from Welgene Inc. The primary antibodies specific for *β*-actin (sc-47778), p21 (sc-756), Bcl-2 (sc-7382), cyclin E (sc-481), and CDK4 (sc-260), and secondary antibodies for anti-rabbit antibody (sc-2357) and anti-mouse antibody (sc-516102) were obtained from Santa Cruz Biotechnology, Inc. Also, the primary antibodies specific for p27 Kip1 (#3686), cytochrome c (#11940), pATR (#2853), Bax (#2772), pCHK2 (#2197), BCL-xL (#2764), pATM (#5883), pBRCA1 (#9009), and pCHK1 (#2348) were obtained from Cell Signaling Technology, Inc. Cyclin D1 (ab6152) antibody was purchased from Abcam. An iNOS antibody (NB300-650) was obtained from Novus Biologicals. Ursolic acid (U6753) was bought in Sigma-Aldrich.

### 2.3. Immunoblotting Analysis

All protein samples were obtained with a RIPA lysis buffer on ice for 20 min. The proteins isolated (100 *μ*g/well) were resolved on SDS-polyacrylamide gels, and then target proteins were identified by immunoblotting assay.

### 2.4. Cell Viability Assay

MTT (Thermo Fisher; M6494) is used for cell viability. Cells of 3 × 10^3^ per well were cultured in a 96-well plate for 24 h. Next, the cells were incubated with DMSO as the vehicle control or diverse concentrations of UA (1–50 *μ*M) for 24 h. The next day, 5 mg/mL of MTT reagent was treated and incubated for 4 h at 37°C. The formazan product was then dissolved in DMSO after being washed with 1X PBS. At a 590 nm wavelength, the absorbance of the formazan product was measured with an Ultra Multifunctional Microplate Reader (Tecan, Durham, NC, USA). All measurements and experiments were conducted in triplicate.

### 2.5. Quantitative Real-Time Polymerase Chain Reaction (qPCR)

Total RNA was isolated using the TRIzol method. Subsequently, a thermal cycler was used to prepare cDNA from the total RNA using a first-strand cDNA synthesis kit (Bioneer) for qRT-PCR. The qPCR was performed using LightCycler 480II (Roche). All reactions were performed three times and normalized to the GAPDH gene, and quantifications were analyzed using the obtained Cp values.

### 2.6. Flow Cytometric Analysis

After cultured cells with prewarmed culturing medium, 1 × 10^6^ cells were resuspended in 1 mL staining buffer containing CM-H2DCFDA (5 *μ*M; Invitrogen; C6827) for cellular ROS, MitoTracker Deep Red (40 nM; Invitrogen; M22426) for mitochondrial membrane potential or MitoSOX (5 *μ*M; Invitrogen; M36008) for mitochondrial ROS. The cells were then incubated in a CO_2_ incubator at 37°C for 30 min. Finally, a cytometric experiment was performed using a FACScalibur™ (BD Biosciences) and then analyzed using a FlowJo software.

### 2.7. Alkaline and Neutral Comet Assay

The Comet Assay Kit (3-well slides; Abcam; ab238544) was used for checking cellular DNA damage. The assay was performed according to the manufacturer's instructions, and cell morphology was observed using a fluorescence microscope (Olympus IX71/DP72).

### 2.8. Cell Cycle Analysis

The DNA content of UA-untreated and UA-treated cells was determined using a BD Cycletest Plus DNA Reagent Kit (BD Biosciences; 340242). Cells of 1 × 10^6^ per sample were used for analyzing DNA content. Then, the cell samples were analyzed using a FACScalibur™ (BD Biosciences) and then analyzed using a FlowJo software.

### 2.9. Apoptosis Detection

The UA-untreated or UA-treated cells were washed with 1X PBS and resuspended in a binding buffer at a concentration of 1 × 10^6^ cells. The apoptosis detection kit (Biolegend; 640914) was purchased, and the apoptosis detection assay was performed according to the manufacturer's protocols. The assay was performed with FACScalibur™ (BD Biosciences), and apoptotic cells were analyzed using a FlowJo software.

### 2.10. Isolation of Mitochondria/Cytosol Fractions

The isolation of mitochondria and cytosol fractions were performed using a mitochondria/cytosol fractionation kit (Abcam; ab65320). The isolation was performed according to the manufacturer's protocols. Immunoblotting assay of cytochrome c was carried out as described above.

### 2.11. Statistical Analysis

Results were expressed as mean ± standard error of the mean. Statistical analyses were conducted using one-way analysis of variance (ANOVA) or Student's *t*-test. One-way ANOVA was also performed using Tukey's post hoc test. The analyses were performed using the SAS 9.3 software.

## 3. Results

### 3.1. UA Suppresses the Propagation in Embryonic CSCs

**Figure 1:**
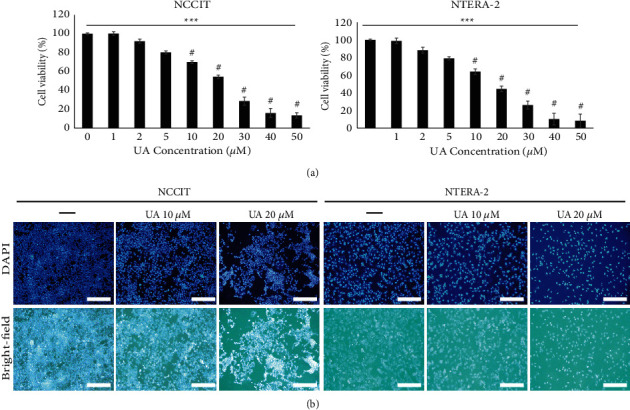
The UA has blocked the proliferation of embryonic CSCs. (a) MTT results showed inhibition of NTERA-2 and NCCIT cell proliferation after treatment with increased UA concentration for 24 hours. The data represent three independent tests. ^#^*p* < 0.001 versus control.  ^*∗∗∗*^*p* < 0.001 (ANOVA test). (b) UA created a nuclear deterioration in the embryonic CSCs. Class comparison microscopy images showing abnormal nucleus formation caused by 24-hour treatment at UA of 10 or 20 µM in NTERA-2 and NCCIT cells. Representing images are displayed (scale bar: 200 *μ*m).

First, we analyzed the ability to inhibit UA proliferation in embryonic CSC function using the MTT assay. We identified a concentration-dependent suppression of viability of cells by UA treatment in CSCs (Figure 1(a)). We used 20 *μ*M UA as an IC_50_ dosage, and a concentration of 10 and 20 *μ*M UA was used for concentration-based studies. These results boosted the UA's ability to target CSCs. We then evaluated the status of embryonic CSCs after UA treatment using DAPI staining and observed a decrease in cell number in UA treatment (Figure 1(b)). The bright-field microscopic analysis also confirmed the inhibition of NTERA-2 and NCCIT cell growth by UA treatment. These results also boosted the UA's ability to prevent the proliferation of CSC.

### 3.2. UA Induces the Generation of Mitochondrial and Cellular ROS

**Figure 2:**
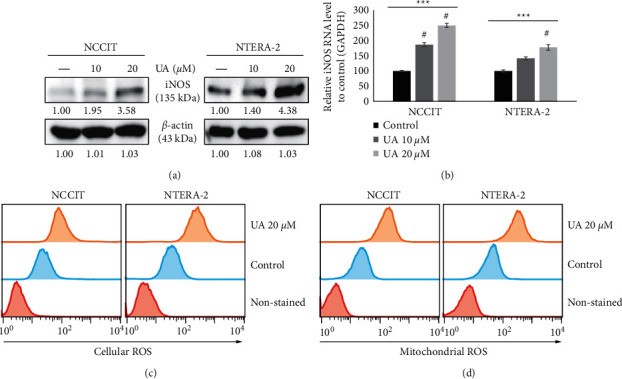
UA produces the formation of cellular and mitochondrial ROS in embryonic CSCs. (a) Immunoblotting analysis of iNOS expression in NTERA-2 and NCCIT cells after treatment for 10 or 20 *μ*M UA 24 h. Exposure rates were measured by densitometry and are standardized in *β*-actin levels. (b) Real-time qPCR analysis showed a symbolic representation of iNOS mRNA in NCCIT cells and NTERA-2 cells. The CP values obtained were normalized by GAPDH mRNA. ^#^*p* < 0.001 versus control.  ^*∗∗∗*^*p* < 0.001 (ANOVA test). (c) Flow cytometry of cellular ROS in NTERA-2 and NCCIT cells after treatment with 20 *μ*M UA for 24 h. Image presentation has shown cells with cellular ROS induction. (d) Flow cytometry of mitochondrial ROS upon 20 *μ*M UA treatment for 24 h in NTERA-2 and NCCIT cells. The graphical image has shown cells with ROS induction in mitochondria.

We determined the UA blockade potential for CSC proliferation in NTERA-2 and NCCIT cells. We assumed that the anti-tumor activity of the UA begins with the production of ROS. To paraphrase this myth, we first examined the patterns of iNOS expression after UA treatment. Our results showed that increased UA concentration regulated the expression levels of iNOS proteins in NTERA-2 and NCCIT cells (Figure 2(a)). To confirm these findings, we analyzed the expression of iNOS mRNA in the embryonic CSC after UA treatment and found the same effect as that observed at the protein level (Figure 2(b)). The launch of iNOS has shown potential ROS production for UA in embryonic CSCs. As expected, we found that the UA had successfully induced the production of ROS in cells (Figure 2(c)) and ROS in mitochondria (Figure 2(d)), suggesting that ROS generation was the cause of anti-tumor activity UA.

### 3.3. UA Induces DDR in Embryonic CSCs

**Figure 3:**
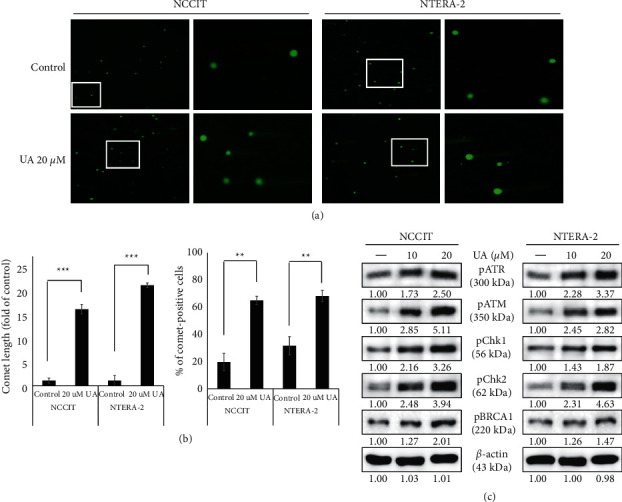
UA induces DDR in embryonic CSCs. (a) The images of a comet assay from fluorescence microscopy at 10x and 40x magnification showing the fragmented DNA migration from the nucleoid body that forms a comet tail in NTERA-2 and NCCIT cells after 24 h treatment with 20 *μ*M UA. (b) Graphical representation of comet length was analyzed as the fold change versus the control and percentage of comet-positive cells with UA treatment in embryonic CSCs. ^*∗∗∗*^*p* < 0.001 and ^*∗∗*^*p* < 0.01 (Student's *t*-test). (c) Immunoblotting analysis of NTERA-2 and NCCIT cells after treatment with 10 and 20 *μ*M UA for 24 h showing manifestations of phospho-histone BRCA1, ATM, ATR, CHK1, and CHK2 proteins. Exposure rates were measured by densitometry and were standardized in *β*-actin levels. Data were received from three different experiments.

Previous results have shown ROS production through UA treatment in the embryonic CSC, which may have been a factor in UA anti-cancer activity. Therefore, we assume that the UA has the potential to produce DDR in NCCIT and NTERA-2 cells. So we checked the UA's ability to recruit DDR using a comet assay to determine duplicate DNA fragmentation, and the results obtained showed that the UA produced DNA duplicate DNA in NCCIT and NTRA-2 cells (Figure 3(a)). We also observed a significant increase in the length of the comet and comet-positive cells in UA-controlled cells compared to untreated control cells (Figure 3(b)). These results demonstrated the ability to import UA-mediated DDR into the embryonic CSC. To confirm this, we examined DDR-binding protein exposure levels and found an increase in exposure to phosphorylated ATMs, ATR, CHK1, CHK2, and BRCA1 when treatment with increased UA concentration focused on NTERA-2 and NCCIT cells (Figure 3(c)). These results suggested that the ATM or ATR serves as the main controller for DDR by the UA.

### 3.4. UA Induces G0/G1 Cell Cycle Arrest

**Figure 4:**
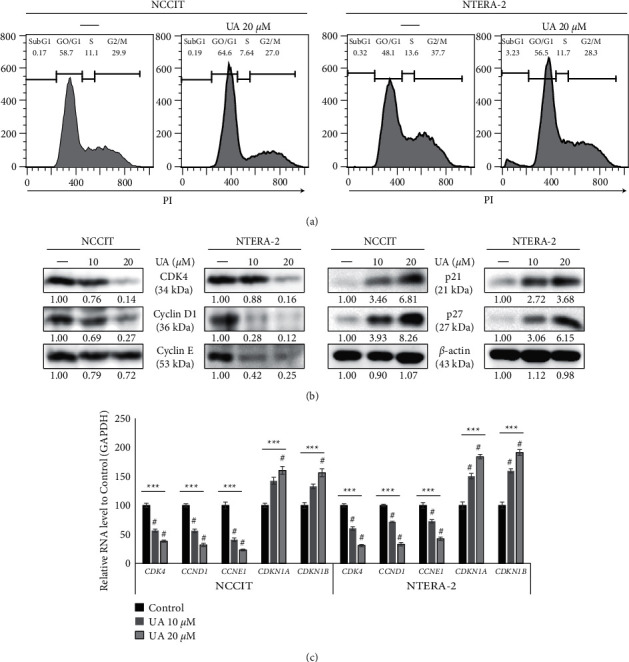
UA induces G0/G1 cell cycle arrest. (a) Flow cytometry using PI showing cell cycle distribution in NTERA-2 and NCCIT cells after 24 h treatment with 20 *μ*M UA. (b) Immunoblotting analysis of NTERA-2 and NCCIT cells after 24 h treatment with 10 or 20 *μ*M UA showing expression of cyclin D1, cyclin E CDK4, p21, and p27 proteins. Exposure rates were measured by densitometry and are standardized in *β*-actin levels. Data were obtained in triplicate. (c) RT-qPCR showing genetic expression of a cell cycle test. References representing CDK4, CDKN1B, CCNE1, CDKN1A, and CCND1 mRNA were displayed; Cp values were normalized to GAPDH mRNA. Controls were set to 100. ^*∗∗∗*^*p* < 0.001 (ANOVA test). ^#^*p* < 0.001 versus control.

Based on previous results, we have shown that the UA has the potential to produce ROS and induce DDR in embryonic CSCs. Therefore, we have analyzed its role in initiating cell cycle arrest. First, cell cycle analysis in embryonic CSCs or without UA treatment using flow cytometry showed binding in the G0/G1 phase of the UA cell cycle to NTERA-2 and NCCIT cells (Figure 4(a)). Slow subgroup formation of subG1 by UA treatment in NTERA-2 cells was also observed, possibly due to the early introduction of apoptosis by the UA. This result showed that the introduction of DDR leads to prolonged cell cycle arrest, and in order to confirm this, we analyzed cell protein test levels for western termination. The results obtained revealed elevated levels of protein-binding proteins, p21, and p27 as well as decreased levels of exposure to cyclin D1, cyclin *E*, and CDK4 proteins (Figure 4(b)). We then validated these results by analyzing the genetic expression pattern responsible for the cell cycle at the mRNA level. The results confirmed the introduction of cell cycle binding by analyzing the expression patterns of CDK4, CDKN1B, CCNE1, CDKN1A, and CCND1 mRNA (Figure 4(c)). These results showed the closure of the cell cycle by the UA in the embryonic CSC and indicated the possible introduction of apoptosis by the UA.

### 3.5. UA Induces Intrinsic Apoptosis in Embryonic CSCs

**Figure 5:**
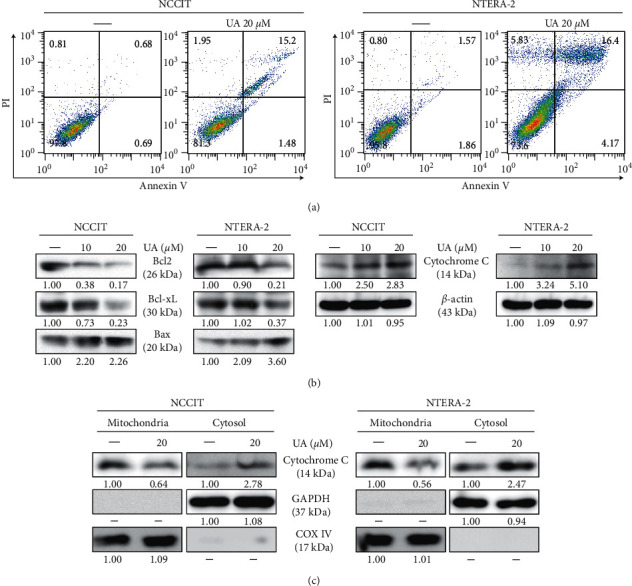
UA induces the intrinsic apoptotic pathway. (a) Fluorescein-conjugated annexin V (annexin V-FITC) versus propidium iodide (PI) staining analysis in NTERA-2 and NCCIT cells showed apoptosis induction after treatment with 20 *μ*M UA for 24 h. (b) Immunoblotting of NTERA-2 and NCCIT cells treated with 10 or 20 *μ*M UA for 24 h showed levels of BCL-2, cytochrome. (c) BCL-xL and BAX expression. Exposure rates were measured by densitometry and were standardized in *β*-actin levels. Data were obtained in triplicate. Immunoblotting of cytochrome protein c in cytosolic and mitochondrial fractions separated from NTERA-2 and NCCIT cells after 24-hour treatment at 20 µM UA. Rate levels of cytosolic cytochrome c were calculated by densitometry and are normalized in GAPDH, and levels of mitochondrial cytochrome c were calculated by densitometry and were standardized in COXIV.

We determined that the UA has the potential to exhibit generation of ROS, DDR, and cell cycle arrest in the embryonic CSC, indicating the potential induction of apoptosis by UA treatment in both NTERA-2 and NCCIT cells. Therefore, we examined the induction of apoptosis by UA treatment in the embryonic CSC using flow cytometry, and the results showed that the UA induced apoptosis in both cells (Figure 5(a)). Based on this introduction of apoptosis, we next investigated the apoptosis method by examining the main controls of apoptosis BCL2-associated *X* (BAX), B-cell lymphoma 2 (BCL-2), B-cell lymphoma-extra-large (BCL-xL), and cytochrome proteins c (Figure 5(b)). Our results showed a reduction in the expression levels of BCL-2 and BCL-xL protein and elevated exposure levels of BAX and cytochrome c upon UA treatment, indicating a possible introduction of the intrinsic pathway mechanism of apoptosis by UA in embryonic CSCs. Because the intrinsic apoptosis is highly dependent on the BAX/BCL-2 ratio, we confirmed the BAX and BCL-2 expression pattern after UA treatment in the embryonic CSC. We found that the UA could induce intrinsic apoptosis in embryonic CSCs. To confirm the release of cytochrome c from mitochondria to the cytosol in UA treatment, we analyzed cytosol-derived proteins and mitochondria and obtained cytochrome c protein by western blotting (Figure 5(c)). The results obtained showed a decrease in the production of cytochrome c in mitochondria in UA treatment, whereas the opposite pattern was observed by exposing cytochrome c to cytosol proteins in both NTERA-2 and NCCIT cells, clearly indicating the expulsion of cytochrome c in cellular mitochondria to proceed for the intrinsic pathway of apoptosis.

## 4. Discussion

The anticancer activity of the selected drug is highly dependent on how it works against cancer cells and their ability to identify CSCs and thus prevent cancer recurrence. Recurrence of cancer is a major complication in various chemotherapeutic drugs because although it effectively suppresses tumor growth, it may fail to target CSCs [28]. This issue makes research on natural anticancer compounds an attractive method because such compounds can be used for long-term exposure as they reduce side effects and can help target both cancer cells and CSCs [29, 30]. There are a few natural compounds that have the potential to work against CSCs [3]. The UA has been verified to perform antitumor activity against different types of cancer cells, thus demonstrating that research on its impact on embryonic CSCs may shed light on its ability to focus on CSCs. Our results have shown that the UA has successfully suppressed NTERA-2 and NCCIT cell proliferation, suggesting that the UA could target cancer cells and CSCs. The morphological analysis also showed the potential for preventing UA proliferation against embryonic CSCs.

A natural compound that can induce DDR and thus bind to the cell cycle and apoptosis in cancer cells can be considered as a potential candidate for further research. Previous studies have demonstrated that UA can induce cell cycle arrest and apoptosis in several cancer types [31–33]. ROS generation tremendously plays an important role in the anticancer activity of a natural compound [34]. We found that UA treatment elevated the expression of iNOS at the transcriptional and translational levels so that iNOS induction leads to ROS generation [7]. Consequently, UA treatment significantly increased cellular ROS and mitochondrial ROS generation, which may indicate UA anticancer activity. We then demonstrated the UA's ability to synthesize DDR with a DNA sequence of double-stranded DNA, and the results showed that the UA could produce DNA strands of double strands in embryonic CSCs. ATM or ATR kinases are considered central controls to DNA damage response and could detect the occurrence of cellular DNA damage [20]. Our results also showed an increase in the expression levels of these DDR kinases, which in turn led to cell cycle arrest and apoptosis. This causes phosphorylation in other substrates such as BRCA1, CHK1, and CHK2 [35, 36]. We noted that UA treatment has successfully influenced the production of cellular and mitochondrial ROS, which activates DDR in embryonic CSCs, which clearly indicates the possibility of cell death by UA treatment.

Induction of DDR could result in prolonged cell cycle arrest and finally apoptosis. Elevated ATM or ATR signaling leads to an increase in genes that suppress the tumor p21 and p27, resulting in the induction of cell cycle arrest. In flow cytometry analysis, the UA treatment improved the p21 and p27 expression levels by lowering the expression levels of CDK4, cyclin D1, and cyclin E, indicating possible cell cycle arrest. Also, embryonic CSCs suggested G0/G1 cell cycle arrest in NTERA-2 and NCCIT cells. The regulation of the expression patterns of cell cycle checkpoints by UA in NTERA-2 and NCCIT cells also supported the induction of cell cycle arrest. These results indicated a possible introduction of apoptosis into the embryonic CSC with UA treatment. The apoptotic pathway is divided into the intrinsic and extrinsic pathways, where mitochondria play a key role in the internal pathway through BAX regulation and downregulation of BCL-2 to enhance cytochrome c release from mitochondria to cytosol for protein activation to activate caspase proteins and then to proceed toward apoptosis [27, 37]. The family proteins of antiapoptotic BCL-2 act as pore antagonists in mitochondrial apoptosis and have the ability to determine the outcome of apoptosis [38]. A proapoptotic BAX contributes to the formation of pores in the cellular mitochondrial membrane. Also, it negatively regulates the expression of BCL-2 to determine whether apoptosis can progress [37]. Our flow cytometry results suggested apoptosis induction by NTERA-2 and NCCI cells. Based on this indication, we analyzed the pathway underlying the apoptosis induction by UA, and our results were consistent with our expectations, as we observed an increase in BAX expression levels and a decrease in BCL-2 and BCL-xL after treatment with UA in embryonic CSCs, which suggested that UA induces mitochondrial apoptosis. We observed an increase in the expression of cytochrome c in the cytosol. We also examined a decrease in control of cytochrome c levels in mitochondria after UA treatment in the embryonic CSC, indicating a possible release of cytochrome c released from mitochondria to the cytosol through the pores created by the BAX/BCL-2 regulation. The cytochrome c directs the cell to the intrinsic apoptosis pathway in mitochondria. Thus, the UA has the potential to induce mitochondrial apoptosis in NTERA-2 and NCCIT cells, in order to target CSCs.

## 5. Conclusion

In conclusion, this study showed that the natural compound of pentacyclic triterpenoid, UA, targets embryonic CSCs by inhibiting the proliferation of NTERA-2 and NCCIT cells. Moreover, the UA clearly induced cellular and mitochondrial ROS generation and DDR against embryonic CSCs. We also demonstrated the induction of G0/G1 phase arrest by UA, which then led to the intrinsic apoptosis in mitochondrial by regulating the expression levels of BAX and BCL-2 as well as cytochrome c levels. Overall, the UA could be regarded as a potential candidate for adjuvant chemotherapy as it could inhibit the recurrence of cancer by targeting CSCs.

## Data Availability

The data used to support the findings of this study are available from the corresponding author upon request.
